# Comprehensive Transcriptomic Profiling of Murine Osteoclast Differentiation Reveals Novel Differentially Expressed Genes and LncRNAs

**DOI:** 10.3389/fgene.2021.781272

**Published:** 2021-11-15

**Authors:** Salman M. Toor, Sachin Wani, Omar M. E. Albagha

**Affiliations:** ^1^ College of Health and Life Sciences, Hamad Bin Khalifa University, Doha, Qatar; ^2^ Rheumatology and Bone Disease Unit, Centre for Genomic and Experimental Medicine, Institute of Genetics and Cancer, University of Edinburgh, Edinburgh, United Kingdom

**Keywords:** osteoclast, osteopororosis, bone resorption, RANK ligand (RANKL), differentiation

## Abstract

Osteoclasts are the sole bone resorbing cells, which undertake opposing roles to osteoblasts to affect skeletal mass and structure. However, unraveling the comprehensive molecular mechanisms behind osteoclast differentiation is necessitated to overcome limitations and scarcity of available data, particularly in relation with the emerging roles of long non-coding RNAs (LncRNAs) in gene expression. In this study, we performed comprehensive and progressive analyses of the dynamic transcriptomes of murine osteoclasts, generated *in vitro*. We compared the total RNA-based transcriptomes of murine bone marrow derived cells with differentiated osteoclasts, while focusing on potentially novel genes and LncRNAs, to uncover critical genes and their associated pathways, which are differentially regulated during osteoclast differentiation. We found 4,214 differentially regulated genes during osteoclast differentiation, which included various types of LncRNAs. Among the upregulated protein coding genes not previously associated with osteoclast are *Pheta1*, *Hagh*, *Gfpt1* and *Nol4*, while downregulated genes included *Plau*, *Ltf*, *Sell* and *Zfp831*. Notably, we report *Nol4* as a novel gene related to osteoclast activity since *Nol4* knockout mice *Nol4* ^*em1(International Mouse Phenotyping Consortium)J*^ exhibit increased bone mineral density. Moreover, the differentially expressed LncRNAs included antisense and long intergenic non-coding RNAs, among others. Overall, immune-related and metabolism-related genes were downregulated, while anatomical morphogenesis and remodeling-related genes were upregulated in early-differentiated osteoclasts with sustained downregulation of immune-related genes in mature osteoclasts. The gene signatures and the comprehensive transcriptome of osteoclast differentiation provided herein can serve as an invaluable resource for deciphering gene dysregulation in osteoclast-related pathologic conditions.

## Introduction

The balance between osteoclast and osteoblast activity can dictate pathogenesis of bone diseases. Osteoclasts are the exclusive bone resorbing cells involved in bone remodeling and resorption, and perform opposing roles to osteoblasts to affect skeletal mass and structure (Teitelbaum and Ross, 2003). Augmented osteoclast activity can lead to bone loss in osteoporosis, inflammatory arthritis and tumor invasion in bone, while osteopetrosis is characterized by increased bone mass and results from attenuation in osteoclast function/recruitment or arrested osteoclastogenesis (Teitelbaum, 2007). Abnormalities in osteoclasts are considered the primary cause of many bone diseases including osteoporosis, the most common bone disorder, and Paget disease of the bone (PDB) in which accelerated osteoclastic bone resorption leads to osteolytic or osteosclerotic bone lesions (Shaker, 2009).

Osteoclasts are multi-nucleated cells belonging to myeloid lineage, and generated via the fusion of monocytes/macrophage precursor cells (Roodman, 1999). Importantly, signaling via tumor necrosis factor (TNF)-family cytokine, receptor activator of nuclear factor (NF)-kappaB ligand (RANKL) and macrophage colony-stimulating factor (M-CSF) are identified as the primary pathways associated with osteoclast differentiation (Asagiri and Takayanagi, 2007). Osteoclast precursors express receptor activator of nuclear factor kappa B (RANK), while its ligand (RANKL) is expressed on osteoblasts/stromal cell precursors, and inhibited by the decoy receptor osteoprotegerin (OPG) (Yasuda et al., 1998). Moreover, various genes including TNF receptor-associated factor (*TRAF*) 6, *NFKB1*, *FOS*, nuclear factor of activated T cells 1 (*NFATC1*), and dendritic cell-specific transmembrane protein (*DC-STAMP*) have been associated with osteoclastogenesis (Asagiri and Takayanagi, 2007), while the tartrate-resistant acid phosphatase (TRAcP) and cathepsin K (CTSK), released by osteoclasts during resorption are identified as specific osteoclast markers (Boyle et al., 2003; Kirstein et al., 2006). Osteoclast functionality in bone resorption is dependent on αvβ3 integrin-mediated induction, binding and polarization, while deficiencies in acidified bone resorptive components disrupt regulated bone remodeling, leading to osteopetrosis. Amplified stimulation of osteoclastogenesis primarily via NFKB signaling can lead to increased osteolysis in osteoporosis (Novack and Teitelbaum, 2008).

Genome-wide screening and the *in vitro* induction of osteoclastogenesis using osteoclast precursor cells and soluble mediators has enabled the identification of molecular mechanisms governing osteoclast differentiation (Asagiri and Takayanagi, 2007). Sequencing techniques and microarray analyses have also contributed to disclosing important genes related to osteoclast differentiation (Cappellen et al., 2002; Day et al., 2004; Nomiyama et al., 2005; Garlet et al., 2008; Coudert et al., 2014; Kim and Lee, 2014; Purdue et al., 2014; Madel et al., 2020). In addition, recent reports have highlighted the roles of long noncoding RNAs (LncRNAs), which have emerged as vital regulators of gene expression, epigenetics and protein translation (Fatica and Bozzoni, 2014), in osteoclast differentiation (Fei et al., 2020; Liu et al., 2020; Yang et al., 2021). However, available data have primarily focused on differences between fully differentiated/generated osteoclasts compared to precursors or focused predominantly on targeted sets of genes, previously linked with osteoclast differentiation/activity. Therefore, a comprehensive insight into the differentially regulated genes and their associated pathways during osteoclast differentiation is warranted.

In this study, we performed sequential and comprehensive transcriptomic profiling of murine osteoclasts generated *in vitro* by comparing the total RNA transcriptomes of bone marrow derived precursor cells with differentiated osteoclasts over different time points. Importantly, we focused on novel genes and LncRNAs, which have not been previously associated with osteoclast differentiation. These progressive analyses provide important insights into the differentially regulated genes and their associated pathways during osteoclast differentiation. Identification of novel gene signatures and LncRNAs associated with osteoclastogenesis can serve as molecular biomarkers for osteoclast differentiation or explored for therapeutic benefits, while the comprehensive transcriptome of osteoclast differentiation provided herein can serve as an invaluable resource for deciphering gene dysregulation in diseases related to osteoclast differentiation/activity.

## Materials and Methods

### Samples

C57 black 6 (CBL/6) mice, with access to adequate nutrition (pelleted RM1; SDS diets, Essex, United Kingdom) and hydration, in a standard animal research facility were utilized in this study. All protocols were performed as per guidelines of the United Kingdom Animals Act of 1986 (Scientific Procedures). Bone marrow (BM) was flushed from long bones of 3-4-month-old CBL/6 mice and cultured in specialized media designed to induce osteoclast differentiation, as described below. All experiments were performed adhering to applicable guidelines and regulations.

### Osteoclast Differentiation

Osteoclast cultures were maintained as described previously (Alonso et al., 2021). Briefly, BM cells from CBL/6 mice were cultured in complete growth media (α-MEM supplemented with 10% fetal calf serum, penicillin/streptomycin and glutamine) and in the presence of soluble M-CSF (Prospec Technology, United Kingdom) at 100 ng/ml to generate bone marrow derived macrophages (BMDM). Non-adherent cells were washed after 48 h, while adherent cells were removed using cell dissociation buffer (Gibco, United Kingdom) and re-cultured in parallel in 6-well plates at a density of 3 × 10^5^ cells per well and in 96-well plates at 1 × 10^4^ cells per well in the presence of M-CSF (25 ng/ml) and soluble RANKL (R&D Systems, United Kingdom) at 100 ng/ml. Cultures in 96-well plates were used to monitor osteoclast differentiation and mature osteoclasts were detected by staining for tartrate-resistant acid phosphatase (TRAcP). Cells with more than three nuclei and positive for TRAcP staining were considered osteoclasts. In addition, the resorptive activity of generated osteoclasts was determined using Osteo Assay plates (Corning, New York, United States), as per manufacturer’s guidelines. Quantification of resorption areas was carried out using ImageJ software (National Institutes of Health, Maryland, United States). Unpaired *t*-test was used to investigate statistical significance between two groups, while one-way Anova test was performed to determine statistical significance between more than two groups (GraphPad Prism, version 9.0; GraphPad Software, California, United States). A *p* value of <0.05 was considered statistically significant.

Cultures in 6-well plates were used to collect RNA for gene expression profiling. Cells were collected at the following time points for gene expression analysis: day 0 (BMDM), day 3 (osteoclast precursor) and day 4 (osteoclast) for subsequent investigations.

### RNA Isolation

RNA was isolated from BMDM cells (Day 0) and differentiated osteoclasts from different time points (Day 3 and Day 4) using GenElute Mammalian Total RNA Kit (Sigma-Aldrich, Missouri, United States) by following manufacturer’s protocol. Integrity and purity of RNA was assessed using Bioanalyzer 2100 (Agilent Technologies, California, United States). A total of nine RNA samples were obtained (representing three biological replicates at Day 0, Day 3 and Day 4).

### Library Preparation and RNA-Sequencing

cDNA libraries were generated from isolated total RNA samples using TruSeq Stranded Total RNA kit with Ribo-Zero Globin (Illumina, California, United States) by following manufacturer’s protocol. Quality-passed libraries were sequenced on NovaSeq6000 system (Illumina) using 100 bp paired-end protocol.

### Data Processing and Differential Gene Expression Analyses

Data were analyzed and illustrated using multiple bioinformatics software under default settings unless otherwise stated. Reads were quality-trimmed using Cutadapt1 (version 1.9. dev2) and those with low quality and short reads (<35 bp) were trimmed along with Illumina TruSeq RNA kit adapters. Reads were aligned to the reference genome (*Mus* Musculus GRmc38) using STAR2 (version 2.5.2b) specifying paired-end reads. Reads were assigned to features of type ‘exon’ in the input annotation grouped by gene_id in the reference genome using featureCounts3 (version 1.5.1). The raw counts table was filtered to remove genes consisting predominantly of near-zero counts, filtering on counts per million (CPM) to avoid artefacts due to library depth. Overall, three biological replicate datasets were generated for each time point (Day 0, Day 3, and Day 4). Abundance data were successively subjected to differential gene expression analyses. Z-scores were calculated from CPM values as described previously (Malone et al., 2011) and heatmaps generated using GraphPad Prism software (GraphPad Software).

Differential gene expression analyses and gene ontology (GO) clustering analyses were performed using Integrated Differential Expression and Pathway (iDEP.92, South Dakota State University, United States) online tool. Raw CPM values were uploaded and computed (min. CPM = 1) to identify and generate various illustrations for gene clustering and differentially expressed genes. Differential expression analysis was performed using the DEseq2 method (Love et al., 2014). PCA and Volcano plots were generated with Log2-fold change (FC) > 2 and false discovery rate (FDR) cutoff <0.05. K-Means clustering were used for performing gene enrichment analyses using GO biological processes pathway database (Ashburner et al., 2000).

## Results

### Transcriptomic Changes in Osteoclast Differentiation

**FIGURE 1:**
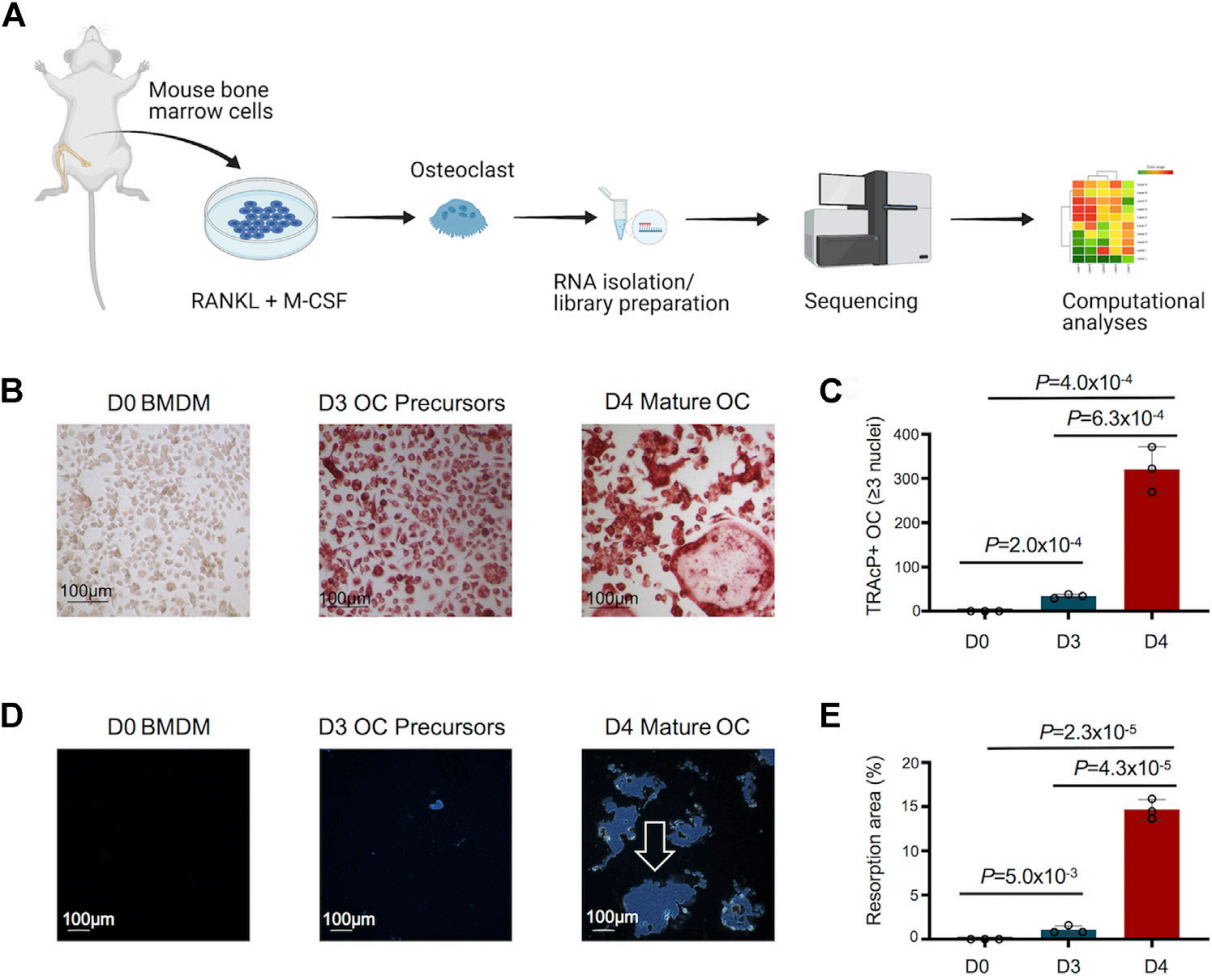
Study design, and morphological and functional changes during osteoclast (OC) differentiation. **(A)** Bone marrow cells flushed from long bones of CBL/6 mice were cultured in the presence of soluble M-CSF and RANKL to induce OC differentiation. Libraries were generated from bone marrow derived macrophages (BMDM) (Day 0) and differentiated osteoclasts at different time points (Days 3 and 4) to perform comprehensive and progressive analyses of the dynamic transcriptomes of murine osteoclasts by RNA-Seq. Various bioinformatics tools were utilized for data analyses/visualization. Schematic representation of the study design is shown. **(B)** The generated OC were detected by staining for tartrate-resistant acid phosphatase (TRAcP). Cells positive for TRAcP staining and with more than three nuclei were identified as OC representative images of TRAcP stained cells show Bone marrow derived macrophages (BMDM) at day 0 (D0), OC precursors at D3 and mature OC at D4 of the culture. **(C)** Scatter/bar-hybrid plot shows the numbers with mean ± standard deviation (SD) of TRAcP+ OC in D0, D3 and D4 comparisons. **(D)** The bone resorption activity of OC was investigated using Osteo Assay. Representative images show cultures from BMDM cells at D0, OC precursors at D3 and mature OC at D4 of the culture. Resorption areas are indicated by white arrow. **(E)** Scatter/bar-hybrid plot shows the differences in percentage resorption area (mean ± SD) in D0, D3 and D4 cultures.

An overview of the study design to decipher the transcriptomic changes during murine osteoclast differentiation *in vitro* is depicted in Figure 1A. The morphological changes in osteoclast differentiation showed the progressive and statistically significant differences in the number of TRAcP+ osteoclasts (≥3 nuclei) formed during differentiation of BMDM cells to osteoclast (Figures 1B,C). Osteoclast precursors exhibit a pre-fusion state whereby the cells cluster together to form multi-nucleated mature osteoclasts (Day 4). In addition, the functional activity of generated osteoclasts was assessed by Osteo Assay (resorptive activity assay), which showed statistically significant, distinct and large resorption areas with osteoclasts compared to osteoclast precursors and undifferentiated BMDM cells (Figures 1D,E). Comprehensive investigations were performed on the differentially regulated genes disclosed during osteoclast differentiation, using stringent criteria and cutoffs as described above.

**FIGURE 2:**
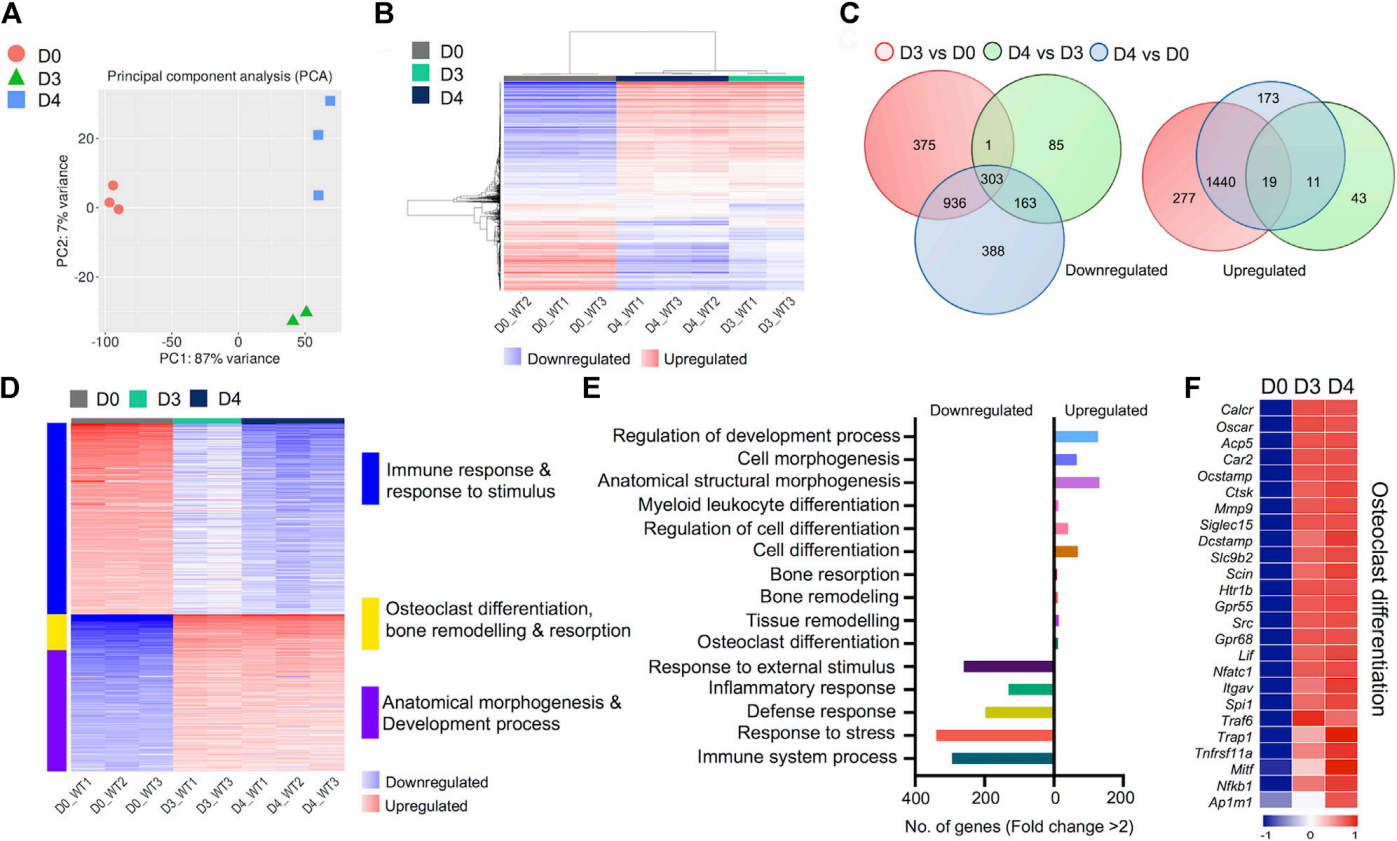
Transcriptomic profiling of osteoclast differentiation. The transcriptomes of bone marrow derived macrophages (BMDM; D0) were compared with osteoclast precursors (D3) and fully differentiated osteoclasts (D4) by performing differential gene expression analyses. **(A)** PCA plot shows variability in gene expression among biological replicates between D0, D3 and D4. **(B)** Heatmap shows hierarchical clustering of gene expression in cells from D0, D3 and D4. **(C)** Venn diagram depicts the total numbers of overlapping differentially regulated (downregulated and upregulated) genes between D0, D3 and D4. **(D)** Heatmap shows gene enrichment and associated pathways in D0, D3 and D4. **(E)** Bar plot shows the numbers of genes corresponding to significantly up/downregulated functional pathways from gene ontology enrichment analyses. **(F)** Heat map shows the gene expression (Z-scores) of osteoclast-related gene panel for confirmation of osteoclast generation.

We generated comprehensive datasets for the transcriptomes of undifferentiated BMDM cells (Day 0) and differentiated osteoclasts on Days 3 and 4 (Supplementary Tables S1-3). One replicate from day 3 failed the quality control measures and was removed from the analysis. PCA analyses showed close proximity of biological replicates for each time point: PC1 showed 87% variance and PC2 showed 7% variance (Figure 2A). Hierarchical gene clustering during osteoclast differentiation showed distinct gene clusters (Figure 2B). Overall, 4,214 differentially regulated genes (2,251 downregulated and 1,963 upregulated) were identified, which showed some overlap between the different timepoint comparisons, primarily between Day 3 versus Day 0 and Day 4 versus Day 0 comparisons (Figure 2C).

Importantly, K-Means clustering analyses revealed the corresponding pathways of gene enrichment observed during osteoclast differentiation (Figure 2D). Our data showed that genes related to immune and inflammatory response, and response to stimulus were downregulated during osteoclast differentiation. Concurrently, genes related to anatomical morphogenesis and developmental process were upregulated in differentiated osteoclasts. Moreover, upregulation of genes related to osteoclast differentiation, bone remodeling and resorption was observed during osteoclast differentiation. Further analyses revealed that the number of upregulated genes related to osteoclast differentiation (n = 12), tissue remodeling (n = 14), bone remodeling (n = 11) and bone resorption (n = 9) was lower compared to the number of downregulated genes associated with immune response (n = 294), response to stress (n = 339), defense response (n = 198), inflammatory response (n = 131) and response to external stimulus (n = 260) (Figure 2E).

Of note, to confirm the *in vitro* generation of osteoclasts, we investigated the expression levels of critical genes known to be expressed in osteoclasts (Teitelbaum and Ross, 2003; Asagiri and Takayanagi, 2007). These selective genes included *Oscar*, *Ocstamp*, *Acp5* (TRAcP), *Ctsk*, *Dcstamp*, *Nfatc1*, *Traf6*, *Trap1* and *Nfkb1*, among others. We found that all genes in our selected panel of osteoclast-related genes were upregulated following induction of osteoclast differentiation (Figure 2F). These confirmatory data provided additional evidence for successful osteoclast generation.

### Differentially Regulated Genes During Early Osteoclast Differentiation

**FIGURE 3:**
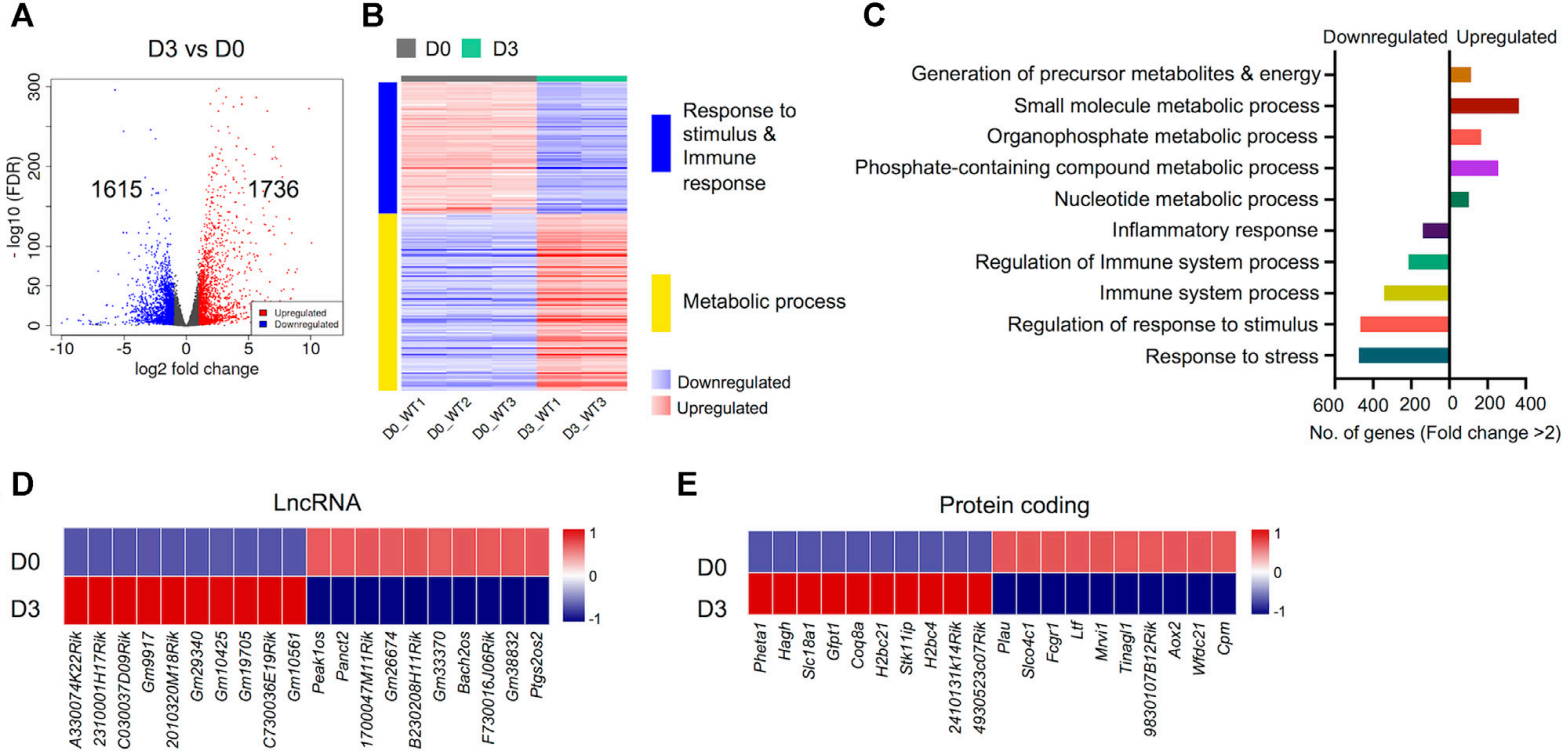
Differentially regulated genes in early osteoclast differentiation. The transcriptomes of osteoclast precursors (D3) were compared with bone marrow derived macrophages (BMDM) (D0). **(A)** Volcano plot represents significantly upregulated (red), downregulated (blue) or genes with unchanged expression levels (gray) between D3 and D0 comparison. **(B)** Heatmap shows gene enrichment and associated pathways in D3 versus D0. **(C)** Bar plot shows the numbers of genes corresponding to significantly up/downregulated functional pathways from gene ontology enrichment analyses. Heat maps show the gene expression (Z-scores) of top differentially-regulated long non-coding RNAs (LncRNA) **(D)** and protein coding genes **(E)** in D3 versus D0 comparison (Log2-FC ≥ 2).

An important aspect of this study was to uncover gene expression profiles during the course of osteoclast differentiation. In this pursuit, we first compared the transcriptomes of early-differentiated osteoclasts (Day 3) with BMDM cells (Day 0; Figure 3). Our data revealed 3,351 differentially regulated genes, of which 1,736 genes were upregulated while 1,615 genes were downregulated in early-differentiated osteoclasts (Figure 3A). Importantly, GO biological process enrichment analyses revealed distinct gene clusters between the transcriptomes of Day 3 versus Day 0 analyses (Figure 3B). Upregulated genes predominantly corresponded to metabolic processes, whereas downregulated genes primarily corresponded to immune and stimulus response. Generation of precursor metabolites and energy- (n = 102), small molecule metabolic process- (n = 256), phosphate containing compound metabolic process- (n = 365) and nucleotide metabolic process-related genes (n = 114) comprised upregulated genes. In contrast, response to stress- (n = 475), regulation of response to stimulus- (n = 467), immune system process- (n = 342) and inflammatory response-related genes (n = 139) comprised downregulated genes during early osteoclast differentiation (Figure 3C).

### Distinct Long Non-coding RNA and Protein Coding Genes Upregulated During Early Osteoclast Differentiation

Apart from protein coding genes, the importance of LncRNAs in the regulation of osteoclast differentiation has been recently described (Fei et al., 2020; Liu et al., 2020; Yang et al., 2021). We compiled a list of differentially regulated LncRNAs during osteoclast differentiation (Supplementary Figure S4). These differentially regulated LncRNAs comprised of different subtypes as listed in Table 1. We found that 35 potentially novel LncRNAs were upregulated of which 20 LncRNAs showed Log2-FC ≥ 2, while 37 LncRNAs were downregulated with 15 LncRNAs showing Log2-FC ≥ 2 during early osteoclast differentiation. The top 20 differentially expressed LncRNAs during early osteoclast differentiation are presented in Figure 3D. These data reflect that these LncRNA genes may have significant roles in osteoclastogenesis and warrant further scrutiny.

**TABLE 1:** Differentially-regulated Long non-coding RNAs (LncRNAs) during osteoclast differentiation.

LncRNA type	Comparison	D3 vs D0		D4 vs D0		D4 vs D3	
	Regulation	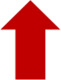	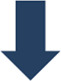	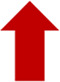	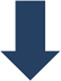	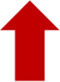	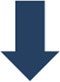
Antisense		40	21	29	28	3	6
LincRNA (long intergenic ncRNA)		35	37	32	40	2	11
Bidirectional promoter LncRNA		12	—	4	—	—	—
Sense intronic		1	—	1	—	—	—
Sense overlapping		1	1	1	—	—	—
**Total differentially-regulated LncRNAs**		**89**	**59**	**67**	**68**	**5**	**17**

In addition, we also identified the top upregulated protein coding genes, which have not been previously reported to be associated with osteoclast differentiation. The top 20 differentially expressed genes based on significance are presented in Figure 3E while, the complete list of potentially novel differentially expressed genes during osteoclast differentiation is provided in Supplementary Table S5. Additionally, the top 10 upregulated and 10 downregulated genes during osteoclast differentiation, based on FC and significance (*p* value), irrespective of novelty, are presented in Supplementary Figure S1.

### Differentially Regulated Genes During Progressive Osteoclast Differentiation

**FIGURE 4:**
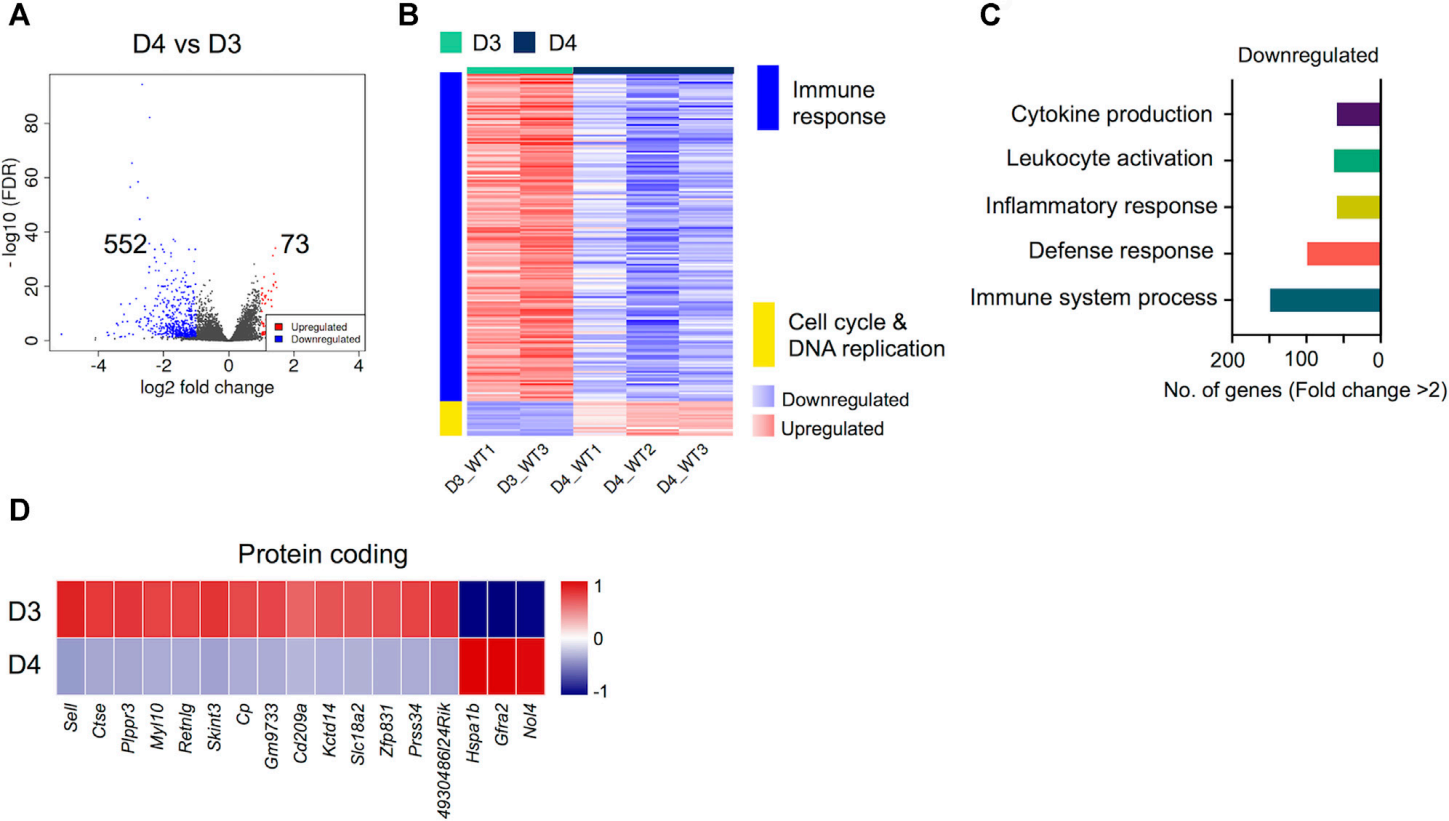
Differentially regulated genes in late osteoclast differentiation. The transcriptomes of fully differentiated osteoclasts (D4) were compared with osteoclast precursors (D3). **(A)** Volcano plot represents significantly upregulated (red), downregulated (blue) or genes with unchanged expression levels (gray) between D4 and D3 comparison. **(B)** Heatmap shows gene enrichment and associated pathways in D4 versus D3. **(C)** Bar plot shows the numbers of genes corresponding to significantly up/downregulated functional pathways from gene ontology enrichment analyses. **(D)** Heat maps show the gene expression (Z-scores) of downregulated and upregulated protein coding genes in D4 versus D3 comparison.

Next, we compared the transcriptomes of early-differentiated osteoclasts (Day 3) with fully differentiated osteoclasts (Day 4). Interestingly, we found that the transcriptomes of committed osteoclasts did not show immense differences with fully differentiated osteoclasts. Overall, only 641 genes showed differential regulation between Day 4 and Day 3 comparison, of which 73 genes were upregulated while 552 genes were downregulated (Figure 4A). Importantly, these differentially regulated genes showed further enrichment in downregulation of immune response-related genes, whereas cell cycle and DNA replication-related processes were upregulated (Figure 4B). The downregulated genes corresponding to immune response-related processes were associated with process such as cytokine production (n = 68), leukocyte activation (n = 73), inflammatory (n = 68) and immune response (n = 112). The potentially novel genes not previously reported in association with osteoclast differentiation/activity are shown in Figure 4D, while the top differentially regulated genes based on FC and significance, irrespective of novelty are presented in Supplementary Figure S1.

### Identifying *Nol4* as a Vital Gene in Mature Osteoclasts

**FIGURE 5:**
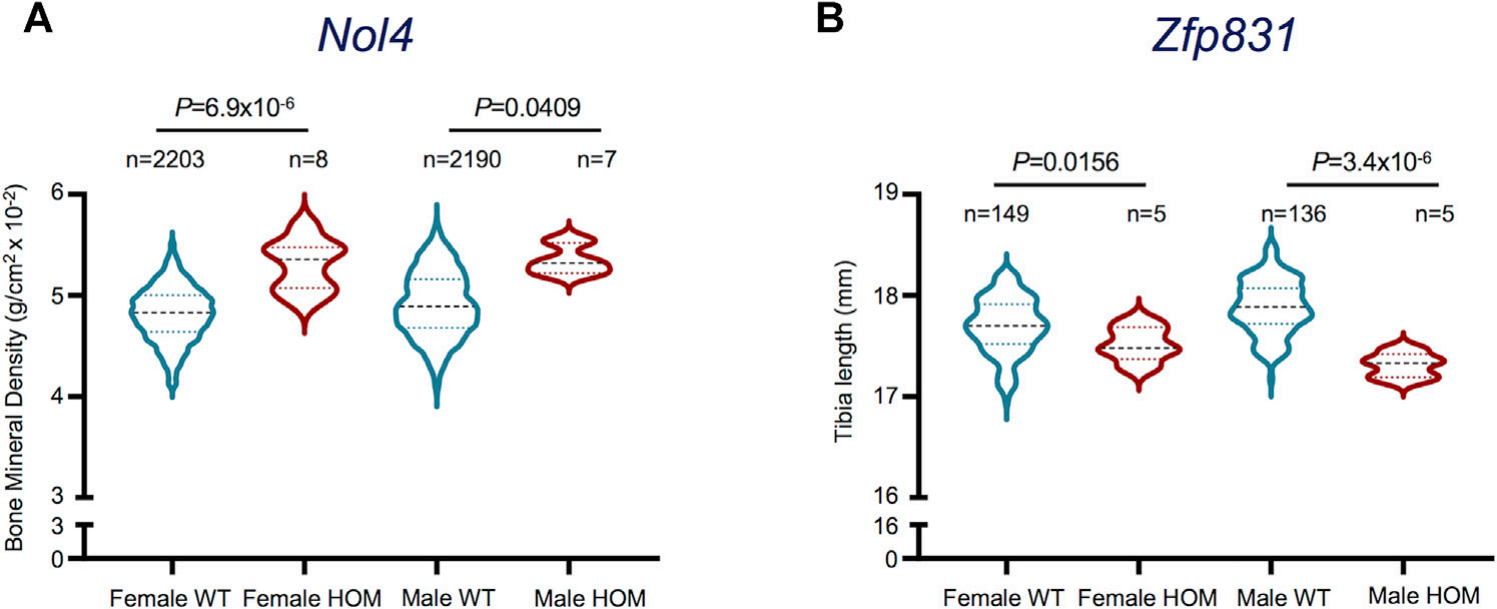
Bone-related phenotypes of Nol4 and Zfp831 knockout mice in the International Mouse Phenotyping (IMPC) datasets. **(A)** Violin plot shows the differences in Bone Mineral Density (excluding skull) in female (n = 8), male (n = 7) homozygote (HOM) mutants for the *Nol4* ^*em1(IMPC)J*^ allele compared to female (n = 2,203), male (n = 2,190) wild type (WT) controls. **(B)** Violin plot shows the differences in Tibia length in female (n = 5), male (n = 5) HOM mutants for *Zfp831* ^*tm1b(KOMP)Wtsi*^ allele compared to female (n = 149) and male controls (n = 136). The interquartile range, minimum and maximum data points, and individual points representing outliers of each dataset are presented. *p* values represent the statistical significance between each comparison [Data taken from the International Mouse Phenotyping Consortium (IMPC) (Dickinson et al., 2016; International Mouse Phenotyping Consortium, 2021)].

The upregulation of selected potentially novel protein coding genes in mature osteoclasts prompted us to explore their prospective roles in osteoclast functionality. Since these genes have not been previously linked with osteoclast generation or activity, we investigated their associated phenotypes in the International Mouse Phenotyping Consortium (IMPC) database (Dickinson et al., 2016; International Mouse Phenotyping Consortium, 2021). Interestingly, we found that Selectin L (*Sell*), Myosin Light Chain 10 (*Myl10*), Resistin-like gamma (*Retnlg*) and Ceruloplasmin (*Cp*) *Signal Regulatory Protein Delta* (*Sirpd/*Gm9733), Zinc Finger Protein 831 (*Zfp831*) and Nuclear Protein 4 (*Nol4*) were previously investigated in relation with bone-related phenotypes. However, only *Nol4* showed statistically significant associations with bone mineral density (BMD) (Figure 5A), while *Zfp831* showed significant associations with tibia length (Figure 5B) in data from IMPC. Performing a body composition (DEXA lean/fat) phenotypic assay on 4,448 mice (male and female) showed that homozygous *Nol4* knockout mic (*Nol4* ^*em1(IMPC)J*^ *,* n = 15) exhibited a significant increase in BMD compared to wild type mice (Figure 5A). While, performing phenotypic assays on *Zfp831* knockout mice (*Zfp831* ^*tm1b(KOMP)Wtsi*^) homozygote mutant mice (n = 10) compared to controls (n = 295) showed a significant reduction in bone (tibia) length (Figure 5B). The downregulation of *Zfp831* in mature osteoclasts in our dataset therefore indicated its potential role in bone development/growth.

## Discussion

The molecular mechanisms behind osteoclast differentiation have been extensively explored. For instance, Capellen *et al.*, used microarray analyses to decipher changes in gene expression during osteoclast differentiation and showed synergy between MCSF and RANKL-induced gene expression (Cappellen et al., 2002). More recently, several potential genetic regulators of osteoclast differentiation have been identified. Nishikawa *et al.*, reported the epigenetic control of osteoclast differentiation via DNA (cytosine-5)-methyltransferase 3A (Dnmt3a) (Nishikawa et al., 2015) and Laha *et al.*, reported regulation of osteoclastogenesis by KLF2 (kruppel-like factor 2 [lung]) via reduction in autophagic cells (Laha et al., 2019). In addition, low-density lipoprotein receptor-related protein1 (LRP1), and COMMD1 were reported as critical regulators of osteoclastogenesis, osteoclast activity and bone mass (Murata et al., 2017; Bartelt et al., 2018), while leucine-rich repeat-containing G-protein-coupled receptor 4 (LGR4) has been identified as another putative receptor for RANKL (Luo et al., 2016).

While these studies revealed important osteoclast-related targets, a comprehensive list of genes provided in our study is of paramount significance in understanding the dynamic changes in gene expression during osteoclast differentiation. Irrefutably, the preexistent available literature related to osteoclast identification and functionality enabled us to confirm the generation of osteoclasts *in vitro*. However, our data revealed the dynamic changes in osteoclast transcriptome during osteoclastogenesis, showing diminishing of genes related to immune processes with concurrent upregulation of genes associated with anatomical morphogenesis and development. Moreover, these genes encoded for important mediators of cell differentiation, structural morphogenesis and bone and tissue remodeling.

The potentially novel genes related to osteoclast differentiation disclosed herein include PH Domain Containing Endocytic Trafficking Adaptor 1 (*Pheta1*), Hydroxyacylglutathione hydrolase (*Hagh*), Solute Carrier Family 18 Member A1 (*Slc18a1*), Glutamine-Fructose-6-Phosphate transaminase 1 (*Gfpt1*), Coenzyme Q8A (*Coq8a*), Solute Carrier Family 14 Member 2 (*Slc14a2*), Whirlin (*Whrn*) and C-Type Lectin Domain Family four Member A (*Clec4a4*), among others. The proteins encoded by these genes are primarily associated with biochemical or signaling pathways at the cellular level and defects in expression may lead to certain disorders. For instance, anomalies in PHETA1/2 have been associated with abnormal bone formation resulting in craniofacial defects (Ates et al., 2020), *HAGH* has been associated with skin, bone and joint infections in Yaws disease (Cheng et al., 2018) and mutations in *GFPT1* have been associated with muscle weakness in congenital myasthenic syndrome (Helman et al., 2019). In addition, histone-related genes H2B Clustered Histone 21 (*H2bc21*) and *H2bc4* were also significantly upregulated during osteoclast differentiation. Epigenetic regulation of bone development and remodeling has been extensively reported (Yi et al., 2019). The upregulation of histone-related genes in osteoclast differentiation indicates chromatin remodeling as a critical step in osteoclast generation.

One of the pivotal novel findings of this study is the upregulation and confirmatory evidence of the potential role of *Nol4* in mature osteoclasts, which has not been reported previously. Investigating bone-related phenotypes of some of the potentially novel genes we recorded showed that selective genes including *Pheta1*, *Hagh*, Plasminogen Activator urokinase (*Plau*), *Sell*, *Retnlg* and *Cp* are related to bone phenotypes in the IMPC database (Dickinson et al., 2016; International Mouse Phenotyping Consortium, 2021). However, only *Nol4* and *Zfp831* showed statistically significant associations with BMD or tibia length, respectively. Importantly, the knockout of *Nol4* led to increase in BMD compared to wild type mice, thereby providing evidence for its role in bone biology. Our differential gene analyses showed upregulation of *Nol4* expression in differentiated osteoclasts, which supports their contribution in affecting BMD in bone resorption and presents *Nol4* as a key gene related to osteoclast functionality. Of note, downregulation of *Zfp831* in mature osteoclasts in our data and association of *Zfp831* knockout with reduced bone (tibia) length in IMPC datasets suggest its potential roles in osteoclast-mediated effects on bone growth and remodeling. However, further investigations are necessitated to fully explore the effects of these genes on bone biology. The protein encoded by *Nol4* is associated with RNA binding and has been identified as a cancer/testis antigen in humans, recently presented as a candidate target in small cell lung cancer (Kim et al., 2021). However, the role of Nol4 in bone-related pathologies remains to be fully explored. Similarly, the protein encoded by *Zfb831* is associated with nucleic acid binding and was downregulated in tumor-infiltrating cytotoxic T lymphocytes (CTLs) compared to CTLs from spleen in a murine transplantable tumor model (Waugh et al., 2016), but its effects in bone-related diseases are not explored.

Accumulating evidences have highlighted the roles of LncRNAs in controlling gene expression to affect cell differentiation and development (Fatica and Bozzoni, 2014). Importantly, in relation with osteoclasts. Liu *et al.*, reported dysregulation in 1,117 LncRNAs in human osteoclasts differentiated from CD14^+^ monocytes *in vitro* (Liu et al., 2020). Fei *et al.*, also reported 204 differentially expressed LncRNAs in male osteoporosis (Fei et al., 2020), while Yang *et al.*, identified 46 differentially expressed LncRNAs between osteoarthritis and osteolysis following total hip arthroplasty, and reported potential roles of specific LncRNA-mRNA pairs in regulating CD8A, CD8B and osteoclastogenesis in these patients (Yang et al., 2021). Moreover, Bu *et al.*, reported the role of LncRNA TSIX in promoting osteoblast apoptosis in particle induced osteolysis (PIO), evident from decreased BMD following implantation, via modulation of the microRNA miR-30a-5p (Bu et al., 2018). These reports rationalize the significance of LncRNAs in osteoclast generation and activity. We performed comprehensive total RNA-based analyses to disclose the differentially regulated LncRNAs during osteoclast differentiation and exclusively reported LncRNA not previously reported or associated with osteoclasts. Importantly, we have highlighted the top 20 novel LncRNAs upregulated during murine osteoclast generation.

Osteoporosis is characterized by attenuated bone strength due to deterioration in bone microarchitecture and reduction in bone mass (Sozen et al., 2017). While various risk factors have been identified for predisposition to osteoporosis, endocrine diseases have also been liked with osteoporosis (Sozen et al., 2017). For instance, in diabetes-associated osteoporosis altered bone metabolism also leads to changes in BMD and has been linked with high osteoclast activity (Kemink et al., 2000; Reni et al., 2016). Importantly, insulin is identified as an essential mediator in osteoclast energy metabolism. Kim *et al.*, investigated the effects on gene expression in insulin-induced osteoclast differentiation and reported that insulin conducts similar roles as RANKL in osteoclast activity (Kim and Lee, 2014). These reports reflect the significance of energy and metabolism in osteoclast activity, which leads to osteoporosis. We found that numerous genes encoding important metabolism/energy-related mediators were significantly increased during early osteoclast generation.

Osteoclast progenitors are essentially immune cells, due to their origins from monocytes/macrophage precursors and may present as innate immune cells within the bone (Wu et al., 2008). Jacquin *et al.,* showed that osteoclast progenitor populations in murine BM comprise CD45R^−^ CD11b^−/low^ populations (Jacquin et al., 2006), while Jacome-Galarza *et al.*, reported that B220^−^CD3^−^CD11b^−/low^CD115^+^CD117^high^ mouse BM cells possess high osteoclastic potential (Jacome-Galarza et al., 2013). Our data showed that osteoclast differentiation deviates their associations with other immunomodulatory cells, evident from downregulation of genes related to immune/inflammatory response and response to stress/stimulus in differentiated osteoclasts. Moreover, we found that the differences between the transcriptomes of fully-committed/differentiated and early-differentiated osteoclasts were predominantly limited to sustained downregulation of genes related to immune cell characteristics. These differentially expressed genes primarily encoded for cytokine production, leukocyte activation and immune response-related functions. Importantly, upregulation of genes related to cell cycle and DNA replication in fully differentiated osteoclasts exhibited the cellular expansion of committed osteoclasts. Of note, induction of bone resorption in inflammatory disorders and immune-related disorders results from the plethora of secreted cytokines, which induce osteoclastogenesis (Adamopoulos, 2018).

Overall, this study disclosed genes and their associated pathways during osteoclast differentiation which can be explored further in successive studies. Importantly, we highlighted the potentially novel genes and LncRNAs in relation to osteoclast differentiation and activity. However, functional studies are necessitated to disclose the roles of specific targets. The comprehensive list of differentially regulated genes provided herein can serve as an expedient tool in osteoclast-related research.

## Data Availability

The datasets presented in this study can be found in online repositories. The names of the repository/repositories and accession number(s) can be found below: NCBI SRA, Accession no: PRJNA769960 (https://www.ncbi.nlm.nih.gov/bioproject/PRJNA769960/).
