# Supplementary material for: Comprehensive Transcriptomic Profiling of Murine Osteoclast Differentiation Reveals Novel Differentially Expressed Genes and LncRNAs
Source: Front Genet. 2021 Nov 15;12:781272. doi: 10.3389/fgene.2021.781272 (PMC8634834; doi:10.3389/fgene.2021.781272)
Supplement: Supplementary file 6 [file Image1.PDF]

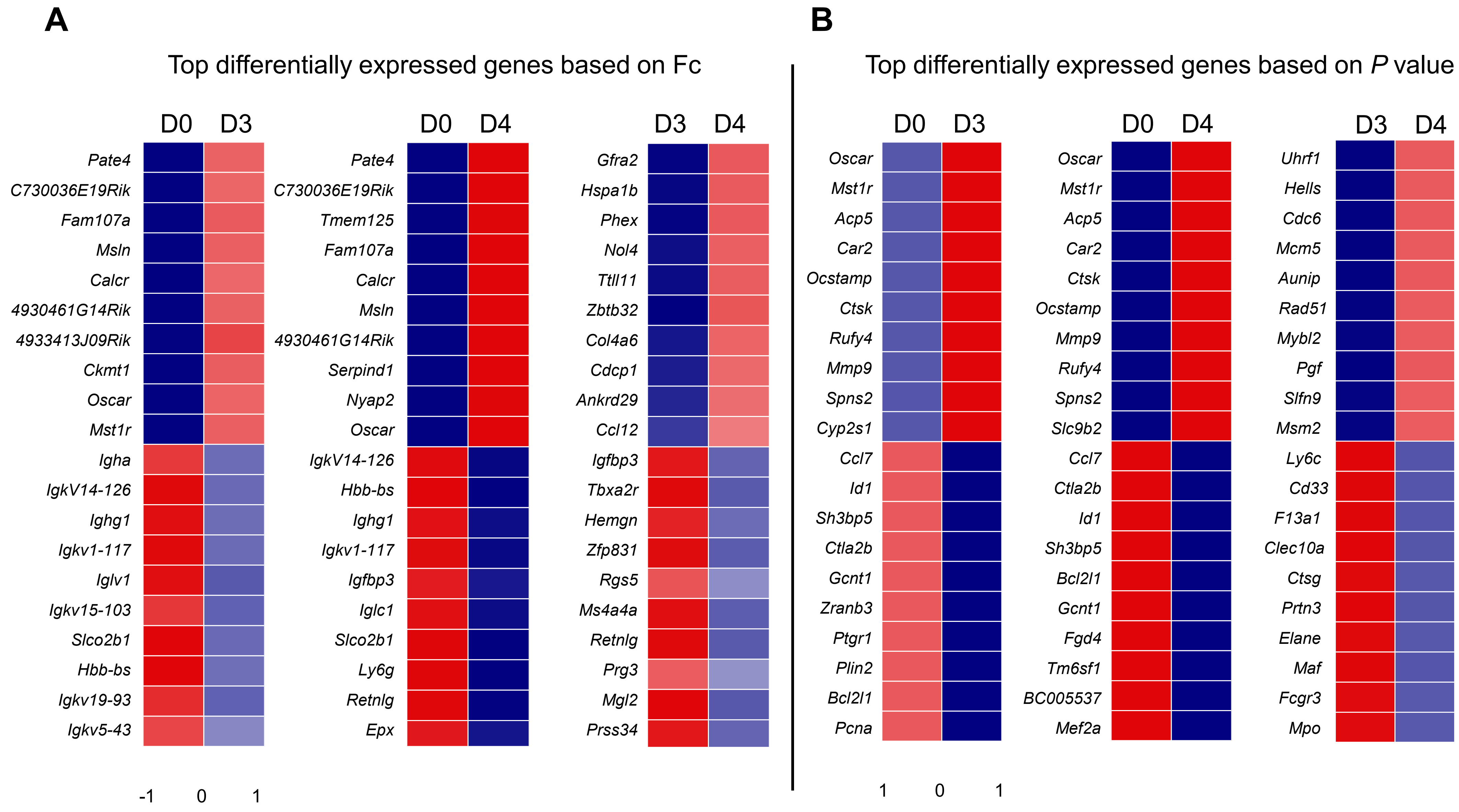

Supplementary Figure 1. Top differentially regulated genes in osteoclast differentiation. The transcriptomes of bone marrow derived macrophages (BMDM) (D0) were compared with osteoclast precursors (D3) and fully differentiated osteoclasts (D4) by performing differential gene expression analyses. Heat maps show the gene expression (Z-scores) of top differentially regulated genes based on differences in fold change (Fc) **(A)** and significance (*P* value) **(B)** between transcriptomes of cells from D0, D3 an D4.
